# Introgressive hybridisation between domestic pigs (*Sus scrofa domesticus*) and endemic Corsican wild boars (*S. s. meridionalis*): effects of human-mediated interventions

**DOI:** 10.1038/s41437-022-00517-1

**Published:** 2022-03-10

**Authors:** Anna Schleimer, Lorraine Richart, Frank Drygala, François Casabianca, Oscar Maestrini, Hannah Weigand, Chantal Schwartz, Michel Mittelbronn, Alain C. Frantz

**Affiliations:** 1 Musée National d’Histoire Naturelle, 25 rue Munster, L-2160 Luxembourg, Luxembourg; 2 Groningen Institute for Evolutionary Life Sciences, University of Groningen, 9700 CC Groningen, The Netherlands; 3 National Center of Pathology (NCP), Laboratoire National de Santé (LNS), 3555 Dudelange, Luxembourg; 4 Luxembourg Center of Neuropathology (LCNP), L-3555 Dudelange, Luxembourg; 5 Department of Oncology (DONC), Luxembourg Institute of Health (LIH), L-1526 Luxembourg, Luxembourg; 6 Doctoral School in Science and Engineering (DSSE), 25 University of Luxembourg (UL), Esch-sur-Alzette, Luxembourg; 7 Association for Nature and Biodiversity (ANB), 60386 Frankfurt am Main, Germany; 8 INRAE Laboratoire de Recherches sur le Développement de l’Elevage, Quartier Grossetti, 20250 Corte, France; 9 Faculty of Science, Technology and Medicine, University of Luxembourg, L-4365 Esch-sur-Alzette, Luxembourg; 10 Department of Life Sciences and Medicine (DLSM), University of Luxembourg, L-4362 Esch-sur-Alzette, Luxembourg; 11 Luxembourg Centre for Systems Biomedicine (LCSB), University of Luxembourg, L-4362 Esch-sur-Alzette, Luxembourg

**Keywords:** Conservation genomics, Genetic hybridization, Structural variation

## Abstract

Owing to the intensified domestication process with artificial trait selection, introgressive hybridisation between domestic and wild species poses a management problem. Traditional free-range livestock husbandry, as practiced in Corsica and Sardinia, is known to facilitate hybridisation between wild boars and domestic pigs (*Sus scrofa*). Here, we assessed the genetic distinctness and genome-wide domestic pig ancestry levels of the Corsican wild boar subspecies *S. s. meridionalis*, with reference to its Sardinian conspecifics, employing a genome-wide single nucleotide polymorphism (SNP) assay and mitochondrial control region (mtCR) haplotypes. We also assessed the reliance of morphological criteria and the melanocortin-1 receptor (*MC1R*) coat colour gene to identify individuals with domestic introgression. While Corsican wild boars showed closest affinity to Sardinian and Italian wild boars compared to other European populations based on principal component analysis, the observation of previously undescribed mtCR haplotypes and high levels of nuclear divergence (Weir’s *θ* > 0.14) highlighted the genetic distinctness of Corsican *S. s. meridionalis*. Across three complementary analyses of mixed ancestry (i.e., STRUCTURE, PCADMIX, and ELAI), proportions of domestic pig ancestry were estimated at 9.5% in Corsican wild boars, which was significantly higher than in wild boars in Sardinia, where free-range pig keeping was banned in 2012. Comparison of morphologically pure- and hybrid-looking Corsican wild boars suggested a weak correlation between morphological criteria and genome-wide domestic pig ancestry. The study highlights the usefulness of molecular markers to assess the direct impacts of management practices on gene flow between domestic and wild species.

## Introduction

Domestication exerts a strong selective pressure on species through genetic processes, such as inbreeding, genetic drift, natural selection to captivity, and artificial selection of desirable traits (Mignon-Grasteau et al. 2005; Price 1984). Over the past 10,000 years, human interventions have led to domesticated species that are morphologically, behaviourally, and genetically distinct from their wild/ancestral conspecifics (Mignon-Grasteau et al. 2005; Zeder 2012). However, particularly before the onset of intense farming practices two centuries ago, domestication rarely occurred in complete isolation from wild conspecifics (Larson and Burger 2013). Indeed, the evolutionary histories of many domesticated species show clear signatures of past introgressive hybridisation, i.e. the exchange of genetic material when fertile hybrids backcross with parental species. Introgressive hybridisation has been reported in cattle (Chen et al. 2018), chickens (Eriksson et al. 2008), geese (Heikkinen et al. 2020), horses (Warmuth et al. 2012), pigs (Frantz et al. 2020), and sheep (Barbato et al. 2017).

The effects of introgressive hybridisation on the morphology, behaviour, and adaptive potential of the introgressed species are largely context-dependent. The black coat of wolves and coyotes is, for instance, a trait that was gained from introgressive hybridisation with domestic dogs and was suggested to confer an adaptive advantage to North American forest wolves (Anderson et al. 2009). Conversely, interbreeding between wild and escaped farmed salmon was linked to changes in phenotypic and life-history traits with potential negative population-level effects in the wild (Glover et al. 2017). Herein lies one of the main concerns about hybridisation between domestic and wild species; introgression dynamics are largely unpredictable and alterations to the local gene pool could induce a loss of adaptation (Bourret et al. 2011), increased invasiveness and population sizes (Fulgione et al. 2016; Le Corre et al. 2020), morphological changes (Anderson et al. 2009; Iacolina et al. 2019), or increased extinction risk (Rhymer and Simberloff 1996; Todesco et al. 2016). In an effort to minimise human interference with the gene pool of wild populations, the default wildlife management recommendation is to prevent hybridisation events between domesticated and wild species (Mcfarlane and Pemberton 2019; Randi 2008). In this context, introgressive hybridisation from domesticated species is often considered to be causing genetic erosion or the loss of genetic integrity in the wild species (Rhymer and Simberloff 1996)

The evolutionary interactions of wild boars (*Sus scrofa*) and domestic pigs (*S. s. domesticus*) are characterised by a complex interplay of domestication, feralisation, and introgressive hybridisation (Frantz et al. 2012; Frantz et al. 2020; Larson et al. 2005,2007; White 2011). Evidence from zoo-archaeological records suggests that pigs were domesticated independently in East Asia, China, (Cucchi et al. 2011) and the Near East, Anatolia, (Ottoni et al. 2013) ~10,500 years before the present (BP). Near Eastern domestic pigs were subsequently introduced into Europe ~8500 year ago, where introgressive hybridisation with European wild boars resulted, over time, in a near-complete disappearance of the original Near Eastern ancestry in the nuclear genomes of European domestic pigs (Frantz et al. 2020; Larson and Burger 2013).

The occurrence of hybridisation with wild boars was tightly linked to then traditional swineherd practices in Europe that allowed pigs to seasonally range freely (Frantz et al. 2020; White 2011). However, with the introduction of modern “improved” pig breeds and industrialisation, most European pig keeping practices transitioned from forest pigs to sty pigs in the 18^th^ century (White 2011). Nowadays, traditional free-range pig keeping is largely limited to a few regions in southern and eastern Europe, e.g., in the Balkans, the Carpathians, Corsica, Sardinia, Sicily and some parts of Greece. Such practices still facilitate gene flow from domestic pigs into the wild boar gene pool and thus impact the genomic make-up of modern-day populations (Iacolina et al. 2018; Koutsogiannouli et al. 2010; Nikolov et al. 2017; Šprem et al. 2014).

Here, we focus on introgressive hybridisation between domestic pigs and wild boars in Corsica and Sardinia, which have recently seen the introduction of differing management approaches. Sardinian authorities were forced to ban traditional free-range pig keeping on the island in 2012 due to the persistent presence of the African swine fever virus since 1978 (Mur et al. 2016). As free-ranging domestic pigs were identified as main reservoir of the disease, a wide-spread eradication programme was initiated (Laddomada et al. 2019). Meanwhile, extensive outdoor farming of pigs is still common practice on Corsica today (Jori et al. 2017). In fact, the Corsican domestic pig breed ‘Nustrale’ was recognised by a PDO (protected designation of origin) in Europe in 2014, promoting the value of the local breed and traditional practices on the island.

The wild boars that are endemic to these Mediterranean islands have been classified as the separate subspecies *Sus scrofa meridionalis*, owing to their phenotypic and biogeographic distinctness (Groves 1989). They differ in their morphology and small size from other European wild boars (Evin et al. 2015) and, based on zoo-archaeological records, originated from the feralisation of prehistoric animals introduced by Neolithic people in the first half of the 6^th^ millennium BCE (Albarella et al. 2006). Using a genome-wide SNP panel, Iacolina et al. (2016) showed that Sardinian wild boars were highly divergent from other European wild boar populations, as well as from domestic pigs, and that the uniqueness of their genetic make-up was not systematically affected by introgression from domestic pigs. Also, a number of private mitochondrial control region sequences has been reported from Sardinia (Scandura et al. 2008), which included sequences from a distinct Italian clade (‘D4’ in Larson et al. 2005; ‘E2’ in Scandura et al. 2008; 2011).

In contrast to Sardinia, there is very little information on the general level of genetic distinctness of Corsican *S. s. meridionalis* (but see Larson et al. 2007). The presence of hybrid morphological traits (e.g. coat colour and shape of the ears; Supplementary Table S1) suggests that hybridisation between Corsican wild boars and domestic pigs is a relatively common occurrence (Jori et al. 2016). From the 1960’s to the 1990’s modern commercial domestic pig breeds (i.e. Large-White, Landrace and Duroc) were crossbred with ‘Nustrale’ to increase litter sizes and growing performances (Casabianca et al. 2000). Given the pig farming practices on the island, there is thus a risk of introgression of modern pig breed genes into the endemic Corsican wild boar gene pool.

The main aim of this study was to fill the current knowledge gaps on the level of genomic distinctness of Corsican *S. s. meridionalis*, particularly in terms of its population divergence from other European wild boar populations and the degree of introgressive hybridisation with local domestic pigs. We hypothesised that putatively “hybrid” wild boars, i.e. wild boars with some domestic morphological traits, had higher proportions of genome-wide domestic pig ancestry compared to morphologically pure-looking wild boars. This part of the study was motivated by the question whether currently employed morphological criteria could be used to confidently identify hybrids and inform management procedures. In comparison with Sardinia, we also tested the hypothesis that human-mediated interventions, i.e. differing pig husbandry management approaches, have had an effect on the level of introgressive hybridisation between domestic pigs and wild boars on Corsica and Sardinia. We hypothesised that the recent ban of free-range pig keeping in Sardinia has resulted in lower domestic pig ancestry in local wild boars, compared to Corsica, where free-range pig keeping is still commonly practiced.

## Materials and methods

Between 2016 and 2017, we collected 56 tissue samples from wild boars that were legally hunted in eight sites in northern Corsica. Based on morphological criteria (Supplementary Table S1, Jori et al. 2016), 38 of these animals were classified by an expert as being wild boars, whereas 18 were considered to be potential hybrids with domestic pigs (we will refer to these as ‘hybrid boar’). In addition, 25 tissue samples from the Nustrale pig breed were collected on ten farms from the same area.

DNA was extracted using an ammonium-acetate-based salting-out procedure (Miller et al. 1988). We used primers pigCTR22L and pigCTR515G (Fickel and Hohmann 2006) and followed the methodology outlined in Frantz et al. (2012) to amplify a 493-bp fragment of the mitochondrial control region (mtCR). Sequence alignment was performed using the MUSCLE procedure (Edgar 2004) imbedded in MEGA v.7 (Kumar et al. 2016). Sequences were collapsed to haplotypes using software COLLAPSE V1.2 (D. Posada; unpublished software). We blasted each distinct mitochondrial haplotype against the pig reference genome Sscrofa11.1 (GCA_000003025.6) to ensure that they corresponded to genuine mitochondrial sequences, rather than nuclear DNA sequences of mitochondrial origin (Schiavo et al. 2017). For each haplotype, we performed a NCBI nucleotide BLAST search to identify identical haplotypes reported in previous work. We used the haplotypes identified in this study in association with the sequences generated by Scandura et al. (2008) to build a haplotype network based on the median-joining method (Bandelt et al. 1999) followed by MP construction (Polzin and Vahdati Daneshmand 2003) using the software NETWORK v4.640 (www.fluxus-engineering.com; unpublished software). Following Frantz et al. (2012), we amplified a 345-bp-long fragment of the melanocortin-1 receptor (*MC1R*) coat colour gene that included the single nucleotide polymorphisms between codon positions 95 and 166 (based on Fang et al. 2009).

A subset of 12 Corsican wild boars and seven domestic pigs from the Nustrale breed, as well as 14 wild boars from Luxembourg (see below), were genotyped using the Porcine SNP60 v2 BeadChip (Illumina Inc.) following manufacturer’s instructions. GenomeStudio 2.0 software (Illumina Inc.) was employed to call genotypes using a custom cluster file to improve call rates. Only autosomal SNPs, mapping to chromosomes 1–18 on the reference genome Sscrofa build 11.1, were retained for analysis. The resulting 60K genotypes were merged with publicly available data from 44 domestic pigs and 60 wild boars sampled in France, Iberia, Italy and Sardinia (Iacolina et al. 2016). The 14 Luxembourg 60K genotypes were generated in the context of a different, unpublished study, but included here to increase the sample size of the wild boar reference data set. Genotypes from 11 Bornean bearded pigs (*S. barbatus*; Yang et al. 2017) were included in analyses requiring an outgroup (i.e. TREEMIX, see below).

Quality control filtering (call rate >90% and missing genotypes <10%) was carried out in PLINK 1.9 (Purcell et al. 2007). The implementation of the KING (Manichaikul et al. 2010) algorithm in PLINK was employed to remove one sample from pairs of closely related (duplicate or 1^st^ degree) samples. The resulting dataset (50K SNP panel set hereafter) was pruned for minor allele frequency (MAF > 0.01) and linkage disequilibrium (LD) in PLINK. SNPs with *r*^2^ > 0.5 were removed from sliding windows of 50 SNPs and with 10 SNPs of overlap using the indep-pairwise function. The pruned dataset is hereafter referred to as 30K SNP panel set and was used in analyses assuming independent SNP loci.

MAFs, observed (*H*_0_) and expected heterozygosity (*H*_*e*_) were estimated from the 50K SNP panel set in PLINK for each domestic pig breed and regional wild boar population. The degree of genetic divergence was estimated using Weir and Cockerham’s *θ* (1984; hereafter Weir’s *θ*) as implementing in the *StAMPP* package (v. 1.6.1; Pembleton et al. 2013) in R (v.3.6.0; R Core Team 2019) using the 30K SNP panel set. Ninety-five percent confidence intervals (95% CI) were estimated based on 100 bootstraps across loci. A principal component analysis (PCA) was performed to investigate ordinal relationships among groups and individuals using the *adegenet* R package (v. 2.1.1; Jombart 2008).

The Bayesian clustering approach as implemented in STRUCTURE (v. 2.3.4.; Pritchard et al. 2000) was employed with the 30K SNP panel set to determine the most likely number of distinct genetic clusters *K* based on the admixture model with correlated allele frequencies. An alternative ancestry prior of *α*=1/*K* as starting value was employed as recommended by Wang (2017). Each estimation comprised an initial 70,000 iterations as burn-in, followed by an additional 200,000 iterations. Ten replicate estimations were conducted for values of *K* ranging from one to 15. The most likely number of *K* was inferred from *Pr*[*X*|*K*], where *X* denotes the data, as described by Pritchard et al. (2000) as well using the ad hoc statistic Δ*K* developed by Evanno et al. (2005). For each *K*, the replicate with the highest *Pr*[*X*|*K*] was plotted with the *pophelper* R package (v.2.3.0; Francis 2017).

The topology of group splits and migration events was inferred from allele frequency variations of the 50K SNP panel set with the TREEMIX algorithm (v. 1.13; Pickrell and Pritchard 2012). *S. barbatus* genotypes were used as outgroup to root the tree. The TREEMIX input file was generated using the *gl2treemix* conversion function in the *dartR* package (v. 1.8.3; Gruber et al. 2018). The maximum likelihood tree was estimated assuming a block size of 20 SNPs (to account for possible LD of adjacent SNPs) with up to 10 migration events *m* over three independent replicate runs. The optimal number of migration edges was inferred employing the linear method implemented in the *optM* package (v. 0.1.3; Fitak 2021). Bootstrap support for splitting and migration events was inferred from 100 bootstrap replicates. Replicate trees were summarised using SumTrees of the DendroPy package (v. 4.5.1; Sukumaran and Holder 2010) and plotted in FigTree (v. 1.4.4.; Rambaut 2018).

The principal components-based algorithm implemented in PCADMIX (v. 1.0; Brisbin et al. 2012) was used to infer local genomic ancestry in Nustrale pigs, Corsican wild boars, and Sardinian wild boars. The method assigns the most likely ancestry proportion along each chromosomal haplotype in admixed individuals in relation to non-admixed reference populations. Local ancestry inferences therefore rely strongly on the chosen reference populations, theoretically representative of the ancestral populations that contributed to the current genomic composition in the admixed individuals. We chose 25 samples from continental wild boar (Italy, *N* = 15; Luxembourg *N* = 10) and 25 samples from domestic pig breeds ‘Large-White’, ‘Duroc’, and Sardinian domestic pigs as putative ancestral reference populations given the putative origin of the insular wild boar populations and documented cross-breeding in the Nustrale breed (Albarella et al. 2007; Lambert-Derkimba et al. 2011). Chromosomal haplotypes were phased in FASTPHASE (v.1.4.8; Scheet and Stephens 2006) using the 50K SNP panel set with default parameters, except for the incorporation of subpopulation labels. Phased haplotypes were pruned for MAF (<0.01) and LD (*r*^2^ > 0.8) in PCADMIX. The window size was set to 20 SNPs. Results were plotted in R using a custom script by Barbato et al. (2017), retaining ancestry designations above a 90% confidence threshold.

The two-layer hidden Markov model implemented in ELAI (v. 1.01; Guan 2014) was employed as an additional approach to infer local ancestry and the structure of haplotypes of Corsican wild boars using the 50K SNP panel set. Unlike PCADMIX, ELAI has the advantage that it works directly with diploid data and, therefore, does not require phased haplotypes. We used the same putative ancestral wild boar and domestic pig reference samples as for the PCADMIX analysis. The two-way admixture estimation was based on five independent expectation maximisation (EM) runs each employing 30 steps (-s 30), two upper-layer clusters (-C 2), and 10 lower-layer clusters (-c10). Mixing was assumed to have occurred over 100 and 1000 generations. Chromosomal admixture proportions were averaged over the five independent EM runs. Individual genome-wide admixture proportions were estimated as weighted averages (weighted by the number of SNPs per chromosome).

Individual genome-wide admixture levels in Corsican wild boars, as estimated in STRUCTURE, PCADMIX, and ELAI, were compared between the proposed “pure” and “hybrid” morphological phenotypes using non-parametric Wilcoxon tests.

SNP data from Corsican wild boars and domestic pigs were screened for potential selection using the PCA-based method implemented in PCAdapt (v.4.3.3; Luu et al. 2017). PCAdapt identifies outlier loci that are excessively related to population structure, making them candidate loci for selection. The method does not require a priori definition of parental source populations and accounts for hierarchical population structuring among samples (Luu et al. 2017). The default method using Mahalanobis distance with *K* = 1 was employed. We focussed on the first principal component as it reflected the divergence among domestic pig and wild boar samples. Outlier SNPs were identified by transforming *P* values into *q* values with a cutoff value of 0.001, ensuring a false discovery rate lower than 0.1% using the R package qvalue (v.2.18; Storey et al. 2019).

## Results

### Mitochondrial control region haplotypes

Sequence analysis of a 472-bp-long-fragment of the mtCR from 81 Corsican suids (38 wild boars, 18 hybrid boars, 25 domestic pigs) revealed a total of 11 different haplotypes, with a total of 25 variable sites consisting of 20 transitions, one transversion and four insertions/deletions (Table 1). A haplotype observed in two domestic pigs was of Asian origin, aligning with Clade A on the median-joining network, while all the other haplotypes were part of the main European E1 clade (Supplementary Fig. 1). The codes of these clades follow the nomenclature by Giuffra et al. (2000). We observed four haplotypes that had not been reported beforehand (although one of them matched a shorter sequence – query cover 83% – from a native Italian pig breed; Table 1, Supplementary Fig. 1). Of these, three were observed exclusively in wild boars (*N* = 34) and one in a wild boar and a domestic pig. One domestic pig carried a haplotype that had only been recorded in domestic pigs (mainly Large White) and a further three pigs carried a haplotype previously observed in a Croatian wild boar (Table 1). The haplotypes observed in the remaining wild boars and domestic pigs had previously been detected in both European wild boar and domestic pig breeds. Altogether 20 wild boars, of which six had been classified as putative hybrids, carried a haplotype also observed in the domestic pig group (Table 1). Despite some overlap, the distribution of haplotypes among pure/hybrid wild boars and domestic pigs suggested differentiation between these *S. scrofa* forms.

**Table 1:** Variable sites in the mitochondrial control region among the 11 haplotypes observed in this study.

Haplotypes^b^	Nucleotide position^a^																									No. of times observed in		
	15493	15503	15523	15541	15544	15559	15566	15572	15573	15574	15581	15589	15594	15617	15658	15677	15703	15715	15730	15742	15759	15826	15841	15879	15888	Wild boar	‘Hybrid’ boar^l^	Domestic pigs
C.H1^c^	T	A	G	A	T	A	G	C	-	A	C	C	A	T	A	T	C	C	A	C	T	C	T	A	C	19	5	0
C.H2^d^	C	-	.	.	.	T	.	.	-	.	.	.	.	.	.	.	.	.	.	.	C	.	.	.	.	2	4	0
C.H3^c^	.	-	.	.	.	T	.	.	A	.	.	.	.	.	.	.	T	.	.	.	C	.	.	.	.	2	2	0
C.H4^e^	.	-	.	.	.	.	.	.	-	.	.	.	.	.	.	.	.	.	.	.	.	.	.	.	.	0	1	0
C.H5^f^	.	-	.	.		T	.	.	-	.	.	.	.	.	.	.	.	.	.	.	C	.	.	.	.	14	4	5
C.H6^c^	.	-	A	.	.	T	.	.	-	.	.	.	.	.	.	.	.	.	.	.	C	.	.	.	.	0	1	1
CD.H7^g^	.	-	.	.	.	T	.	.	-	.	.	.	.	C	.	.	.	.	.	.	C	.	.	.	.	1	1	9
CD.H8^h^	.	-	.	.	.	T	.	.	-	.	.	.	.	C	.	.	.	.	.	.	.	.	.	.	.	0	0	4
CD.H9^i^	.	-	.	.	C	.	A	-	-	.	T	T	G	C	.	C	.	.	G	T	.	T	C	.	T	0	0	2
CD.H10^j^	.	-	.	.	.	T	.	.	-	.	.	.	.	.	.	.	.	T	.	.	.	.	.	.	.	0	0	1
CD.H11^k^	.	-	.	.	.	T	.	.	A	.	.	.	.	C	.	.	.	.	.	.	C	.	.	.	.	0	0	3

The control region was sequenced in 56 Corsican wild/hybrid boar and 25 Corsican domestic pigs. Dots (.) and dashes (−) indicate matches and gaps, respectively, with the master sequence (C.H1). Boars were classified as putative ‘hybrids’ based on phenotypic traits in Supplementary Table 1.

^a^Nucleotide positions are initially numbered according to the complete pig mtDNA reference sequence (Ursing and Arnason, 1998), but change later due to insertions/deletions;

^b^Genbank accession numbers MH746786-MH746796;

^c^Sequence without complete match on Genbank;

^d^Sequence without complete match on Genbank, but matched a shorter sequence (query cover 83%) from a native Italian pig breed (e.g. EU362554);

^e^Observed previously in wild boars (e.g. Genbank accession number AY884672) and domestic pig breeds (e.g. AY884778);

^f^Observed previously in wild boars (e.g. KC771440) and domestic pig breeds (e.g. JQ273478);

^g^Observed previously in wild boars (e.g. JQ273229) and domestic pig breeds (e.g. JQ273265);

^h^Observed previously in Corsican wild boars (e.g. AY884681) and domestic pig breeds (e.g. JQ273480);

^i^Sequence of Asian origin;

^j^Observed previously only in domestic pig breeds (e.g. JQ273465, JQ27348).

^k^Observed previously only in a Croatian wild boar (MF196767).

^l^Putative hybrid boar based on phenotypic traits outlined in Supplementary Table 1.

### Coat colour gene (MC1R) diversity

Sequence analysis of a 345-bp fragment of the *MC1R* gene in 76 samples (35 wild boar, 17 hybrid boar, 24 domestic pigs, additional five samples did not pass quality control) revealed the presence of five different alleles. While the majority of the wild boar (32 individuals; 91%) were homozygous for the wild type 0101/E + allele, this was only the case for four hybrid boars (24%; Table 2). All the remaining wild/hybrid boar and domestic pigs were characterised by one or two copies of a dominant black allele of European origin (0301/ED2). In addition, we identified a dominant black allele of Asian origin (0201/ED1) in one hybrid boar and European alleles for black spotting and recessive red in the domestic pigs (Table 2).

**Table 2:** Summary of alleles observed at the *MC1R* coat colour locus in 76 domestic pigs and wild/hybrid boars from Corsica.

*MC1R*		No of animals		
Allele 1	Allele 2	Wild boar	Hybrid boar	Domestic pig
0101/*E*^*+*^	0101/*E*^*+*^	32	4	0
0101/*E*^*+*^	0301/*E*^*D2*^	3	9	0
0301/*E*^*D2*^	0201/E^D1^	0	1	0
0301/*E*^*D2*^	0301/*E*^*D2*^	0	3	21
0301/*E*^*D2*^	0401/*e*	0	0	1
0301/*E*^*D2*^	0503/*E*^*P*^	0	0	2

*Allele 0101/E*^*+*^ European wild type, *0201/E*^*D1*^ Asian dominant black, *0301/E*^*D2*^ European dominant black, *0401/e* European recessive red, *0503/E*^*P*^ European black spotting; following nomenclature of Fang et al. (2009).

### SNP summary statistics

**Fig. 1:**
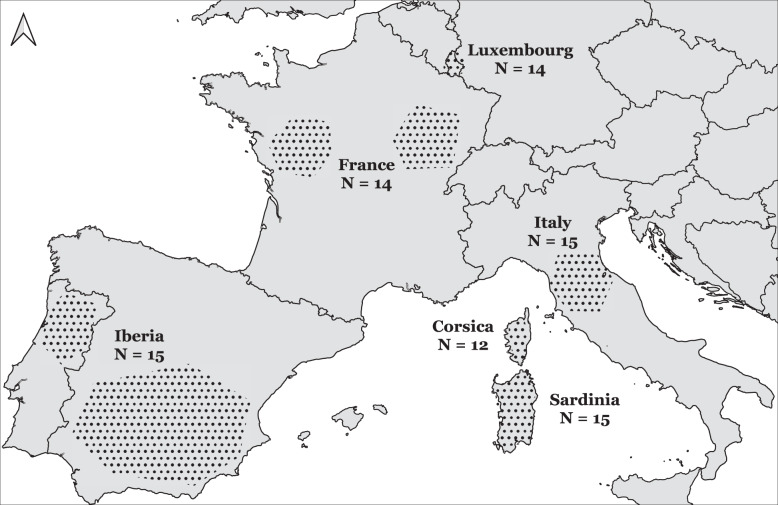
Geographical distribution of wild boar samples. Approximate regional locations and sample sizes *N* of *Sus scrofa scrofa* samples in continental Europe and of *S. scrofa meridionalis* in Corsica and Sardinia.

After merging data libraries and filtering for missing data, genotypes from 131 unrelated individuals genotyped at 48 222 autosomal SNPs (50K SNP panel set) were retained. The data set consisted of 85 wild boars (Fig. 1) and 46 domestic pigs (Table 3), with a high genotyping rate of 99.5%. The LD- and MAF-pruned data set was reduced to a SNP panel set of 28 089 SNPs (30K SNP panel set). Average expected and observed heterozygosity was estimated to be lower in wild boar (*H*_*e*_ = 0.253, *H*_*o*_ = 0.196) than domestic pig samples (*H*_*e*_ = 0.350, *H*_*o*_ = 0.273; Table 3). Similarly, wild boar samples were characterised by lower MAF (average MAF = 0.148), compared to domestic pig samples (MAF = 0.191). An exception to this trend was the Mora Romagnola breed, with the lowest levels of heterozygosity and MAF of all samples.

**Table 3:** Sample sizes (*N*) and summary statistics of regional wild boar (WB) populations and domestic pig breeds.

	*N*	*H*_*e*_	*H*_*o*_	MAF
WB Corsica	12	0.213	0.220	0.149
WB Sardinia	15	0.194	0.162	0.138
WB Italy	15	0.198	0.183	0.141
WB France	14	0.217	0.215	0.155
WB Luxembourg	14	0.211	0.207	0.152
WB Iberia	15	0.217	0.197	0.156
Nustrale	7	0.306	0.320	0.220
Sardinian Feral	8	0.329	0.326	0.240
Nera Siciliana	7	0.258	0.261	0.185
Mora Romagnola	6	0.126	0.152	0.085
Duroc	10	0.249	0.258	0.179
Large White	8	0.323	0.303	0.237

*H*_*e*_ expected heterozygosity, *H*_*o*_ observed heterozygosity, MAF minor allele frequency.

### Population differentiation

The overall degree of population divergence between our study populations was estimated to be Weir’s *θ* = 0.102 (95% CI 0.1–0.103, Table 4). The pairwise estimate of genetic divergence between Corsican wild boar and the local Nustrale breed was estimated at Weir’s *θ* = 0.148 (95% CI 0.145–0.151), which was lower than the genetic divergence estimated among Corsican and Sardinian wild boars (Weir’s *θ* = 0.168, 95% CI 0.164–0.172) and Corsican and Italian wild boars (Weir’s *θ* = 0.187, 95% CI 0.183–0.191). The genetic divergence estimates among wild boar populations ranged between Weir’s *θ* = 0.107, (95% CI 0.102–0.110; France–Luxembourg) and Weir’s *θ* = 0.209 (95% CI 0.205–0.213; Luxembourg–Sardinia). The lowest degree of pairwise genetic divergence was estimated between Sardinian feral pigs and the Large White breed (Weir’s *θ* = 0.030, 95% CI 0.028–0.031). The divergence between the Nustrale and Large White breeds was similarly one of the lowest observed in the study (Weir’s *θ* = 0.087, 95% CI 0.084–0.090). The highest degree of divergence included the Mora Romagnola breed.

**Table 4:** Pairwise genetic divergence between wild boar (WB) populations and domestic pig breeds estimated as Weir and Cockerham’s (1984) *θ* (below diagonal) with 95% confidence intervals (above diagonal).

	WB Co	WB Sa	WB It	WB Fr	WB Lu	WB Ib	NU	SA	NS	MR	Duroc	LW
WB Co		0.164–0.172	0.183–0.191	0.196–0.204	0.169–0.176	0.202–0.212	0.145–0.151	0.179–0.186	0.188–0.198	0.411–0.419	0.326–0.335	0.227–0.234
WB Sa	0.168		0.130–0.137	0.171–0.176	0.205–0.213	0.176–0.182	0.202–0.211	0.179–0.186	0.179–0.185	0.409–0.417	0.326–0.335	0.239–0.245
WB It	0.187	0.134		0.147–0.152	0.182–0.190	0.154–0.160	0.200–0.208	0.177–0.183	0.181–0.188	0.404–0.412	0.320–0.329	0.232–0.238
WB Fr	0.200	0.174	0.150		0.102–0.110	0.106–0.111	0.175–0.182	0.150–0.155	0.162–0.168	0.376–0.383	0.293–0.301	0.199–0.205
WB Lu	0.173	0.209	0.186	0.107		0.147–0.154	0.152–0.159	0.181–0.189	0.195–0.203	0.400–0.410	0.318–0.327	0.227–0.235
WB Ib	0.207	0.179	0.157	0.109	0.151		0.175–0.183	0.151–0.157	0.161–0.167	0.369–0.377	0.289–0.296	0.201–0.206
NU	0.148	0.207	0.204	0.178	0.156	0.179		0.056–0.062	0.096–0.104	0.325–0.334	0.226–0.234	0.084–0.090
SA	0.182	0.182	0.180	0.153	0.185	0.154	0.058		0.065–0.069	0.286–0.294	0.177–0.183	0.028–0.031
NS	0.193	0.182	0.184	0.166	0.200	0.164	0.101	0.067		0.340–0.349	0.241–0.249	0.112–0.118
MR	0.415	0.413	0.407	0.379	0.405	0.373	0.329	0.290	0.345		0.398–0.408	0.320–0.327
Duroc	0.331	0.331	0.324	0.297	0.323	0.293	0.230	0.181	0.245	0.404		0.229–0.236
LW	0.230	0.242	0.234	0.202	0.232	0.204	0.087	0.030	0.115	0.324	0.233	

*Co* Corsica, *Sa* Sardinia, *It* Italy, *Fr* France, *Lu* Luxembourg, *Ib* Iberia, *NU* Nustrale, *SA* Sardinian feral pig, *NS* Nera Siciliana, *MR* Mora Romagnola, *LW* Large White.

**Fig. 2:**
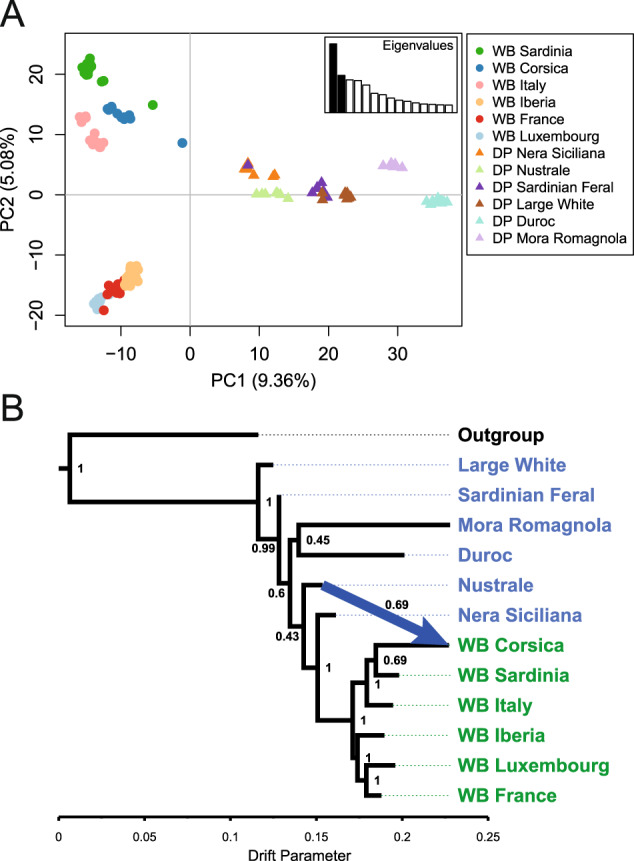
Genomic differentiation and relationship among *Sus scrofa* samples. **A** Principal component (PC) analysis of wild boar (WB) and domestic pig (DP) 30K SNP genotypes; **B** Topology of population splits of domestic pig breeds (blue) and wild boars (green) as inferred in TREEMIX; the node label denotes bootstrap support; the arrow denotes a migration edge from Nustrale to Corsican wild boars with 69% bootstrap support; *S. barbatus* genotypes were employed as outgroup to root the tree.

In the PCA, the first principal component accounted for 9.36% of the variance and discriminated between wild boars and domestic pigs, with the Mora Romagnola and Duroc breeds clustering away from the remaining domestic pig clusters (Fig. 2A). The second PC, accounting for 5.08%, separated wild boars into two clusters; the first one including Sardinian, Corsican, and Italian wild boar and the second one composed of Iberian, French, and Luxembourgish wild boars. Among the Corsican and Sardinian wild boars were two outlier individuals that likely represented recent wild boar/domestic pig hybrids (Fig. 2A).

**Fig. 3:**
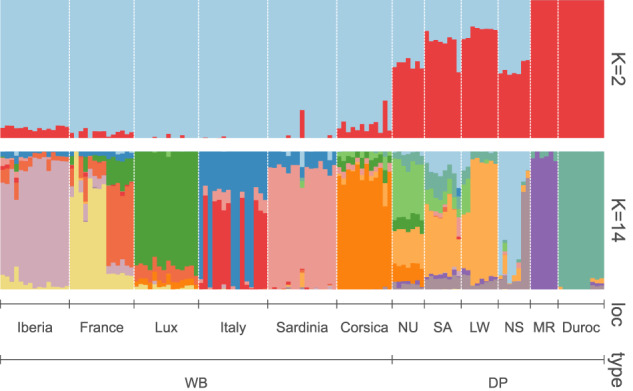
Clustering solutions inferred in STRUCTURE from the 30K SNP panel set at *K* = 2 and *K* = 14 for six regional wild boars (WB) populations and six domestic pig (DP) breeds. NU Nustrale, SA Sardinian feral pig, LW Large White, NS Nera Siciliana, MR Mora Romagnola.

Bayesian ancestry inference, as implemented in STRUCTURE, indicated strong hierarchical clustering (Fig. 3). Evanno’s *ΔK* method suggested *K* = 2 as the most likely number of clusters, separating wild boars from domestic pigs (Supplementary Fig. 2). Corsican wild boars showed the highest levels of admixture among all sampled wild boars at *K* = 2, with an averaged inferred ancestry of 90.8% wild boar and 9.2% domestic pig. In comparison, the inferred domestic pig ancestry in Sardinian wild boars was estimated at 1.6%. The highest *Pr*[*X*|*K*] was observed at *K* = 14, separating wild boars according to geographical regions and domestic pigs according to breeds (Fig. 3, Supplementary Fig. 2). The Nustrale and Nera Siciliana breeds both showed high levels of admixture levels at *K* = 2 and K = 14.

The topology of population splits and migration events inferred in TREEMIX reflected the results of the PCA and STRUCTURE, with Mora Romagnola and Corsican wild boar showing the strongest signals of genetic drift among domestic pigs and wild boars, respectively (Fig. 2B). Based on simple exponential and non-linear least squares modelling, a single migration edge from the Nustrale breed to Corsican wild boars was retained as the optimal number of migration edges with 69% bootstrap support (Fig. 2B).

### Inference of local genomic ancestry

**Fig. 4:**
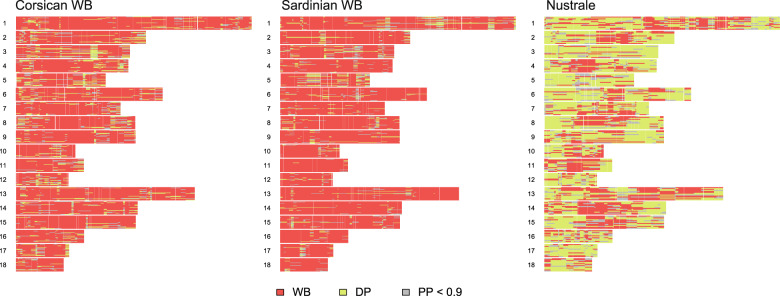
Graphical representation of PCADMIX results for Corsican and Sardinian wild boars (WB) and the domestic pig breed Nustrale for chromosomes 1–18 (horizontal bands). Each line within a chromosomal band represents a haploid individual. The horizontal axis represents chromosome size in base pairs. Genomic regions are coloured according to the most likely ancestry assigned by PCADMIX (i.e., WB in red or DP in yellow). Regions with a posterior probability below 0.9 are shown in grey. Plots generated with R code provided by Barbato et al. (2017).

PCADMIX indicated that Corsican wild boars showed a significantly larger mean proportion of domestic pig ancestry (9.32%) compared to Sardinian wild boars (average 5.49%). Blocks of domestic pig ancestry were distributed across all chromosomes, with only a few blocks showing converging ancestry across all individual haplotypes (Fig. 4). Wild boar ancestry accounted for 32.51% in the Nustrale breed samples.

Assuming different lengths of mixing generations (mg) in ELAI, the amount of inferred domestic pig ancestry was estimated at 6.47% (100 mg) and 10.01% (1000 mg) in Corsican wild boars. In comparison, Sardinian wild boar samples were inferred to comprise only 2.42% (100 mg) and 5.47% (1000 mg) domestic pig ancestry. Nustrale breed samples showed high levels of inferred wild boar ancestry of 44.32% (100 mg) and 40.23% (1000 mg).

**Fig. 5:**
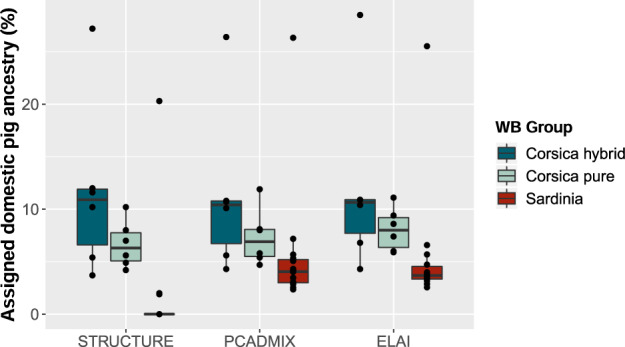
Comparison among three methods used to infer the proportion of domestic pig ancestry in Corsican (blue) and Sardinian (red) wild boars. Corsican wild boars were categorised into putative pure and hybrid individuals based on morphological traits. Black dots show individual sample points.

**Fig. 6:**
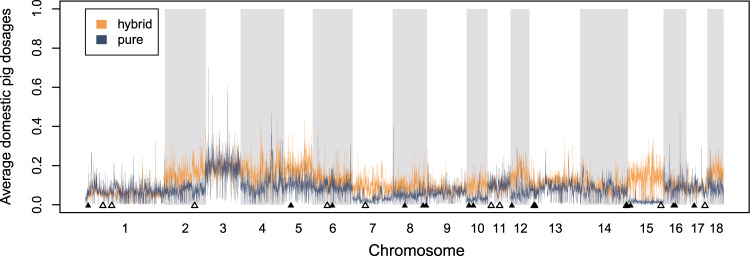
Graphical representation of ELAI results (assuming 1000 mixing generations) for morphologically pure and hybrid Corsican wild boars. The y-axis shows the average domestic pig dosages for each SNP of the 50K SNP panel set (x-axis). Inferred domestic pig dosages were averaged across individuals and across the five independent runs. Triangles show the position of protein-coding (black) and non-coding (white) outlier loci as identified by PCAdapt.

Across all three approaches (i.e., STRUCTURE, PCADMIX, ELAI), individual-level differences in estimates of local ancestry proportions were observed among Corsican wild boars (Fig. 5). Corsican wild boars had been categorised by an expert into putative “pure” and “hybrid” individuals based on external phenotypic characteristics. While the inferred amount of domestic pig ancestry was on average 4.3% larger in the putative hybrid individuals (average 11.7%, 95% confidence interval (CI) 8.07–15.2%) than the putative pure wild boar individuals (average 7.3%, 95% CI 6.3–8.39%), this difference was not statistically significant (p > 0.05; based on Wilcoxon’s non-parametric test given small sample sizes). In fact, some of the morphologically pure-looking wild boars were estimated to hold 10% domestic pig ancestry, while the individual with the lowest levels of inferred domestic pig ancestry was categorised a hybrid based on morphological traits. The two outliers from the PCA (Fig. 2A) also appeared as outliers in all three ancestry inference methods (Fig. 5), potentially representing backcrossed hybrids with ~25% remaining domestic pig ancestry. Average domestic pig dosages differed across chromosomes between morphological groups, with distinctly higher levels of domestic pig dosages on chromosomes 2, 7, 10, and 15 in morphologically hybrid individuals (Fig. 6). Concurrently, both pure and hybrid individuals showed elevated domestic pig dosages on chromosome 3.

PCAdapt identified 30 outlier SNPs that were significantly associated with the differentiation among Corsican domestic pigs and wild boars at a false discovery rate at 0.1% (Supplementary Fig. 3). Seventeen of these loci are associated with known protein-coding genes and may be subject to selective pressures (Supplementary Table S2). These included genes associated with spermatogenesis (*SPATA17, SPATA18*), visual perception (*CRB1*), and hearing development (*LRIG3*). The outlier loci were distributed across 14 chromosomes and their position did not coincide with areas of increased estimated domestic pig ancestry proportions (Fig. 6).

## Discussion

### The genetic distinctiveness of Corsican *S. s. meridionalis*

This study aimed to fill the knowledge gaps surrounding the genetic distinctiveness of Corsican wild boars, with respect to its Sardinian conspecifics and other European wild boar populations. In line with Sardinian wild boars (Scandura et al. 2008), all mtCR haplotypes observed in the present study were assigned to the main European E1 clade, which is composed of the majority of European wild boars and domestic pigs (Giuffra et al. 2000). Haplotypes from the rarer E2 clade, which have so far only been reported in Italian and Croatian wild boars (Larson et al. 2007; Scandura et al. 2008), were not observed in Corsican wild boars. Scandura et al. (2008) reported the presence of E2 haplotypes in two Sardinian museum specimens. Larson et al. (2007) reported the presence of a mtCR haplotype of Near Eastern origin both in a historical (15^th^ century) and a contemporary Corsican *S. scrofa*. Here, a mtCR haplotype belonging to the Asian clade (A) was only observed in two domestic pigs. However, it cannot be excluded that clade A or E2 haplotypes also occur at low frequency in Corsica and remained undetected due to insufficient sampling intensity. Conversely, over half of the sampled Corsican wild boars carried mtCR haplotypes that had not previously been described in Sardinian or other wild boar populations, indicative of a clear divergence of Corsican wild boars from other European wild boar populations.

Nuclear markers provided complementary insights into the genetic distinctiveness of Corsican wild boars. The principal component analysis and TREEMIX divided the wild boars into a western (Iberia, France, Luxembourg) and a southern (Italy, Corsica, Sardinia) cluster, in agreement with a postglacial demographic expansion from an Iberian refugium, and the Alps hindering a northward range expansion (Scandura et al. 2008). The lack of recent gene flow between Corsican and Sardinian and Corsican and Italian wild boars has resulted in genetic divergence estimates (i.e. Weir’s *θ*) comparable to the levels of divergence estimated among continental wild boar populations, which was also reflected in clear clustering in the Bayesian clustering analysis.

TREEMIX suggested that Corso-Sardinian wild boars shared a common ancestor with Italian wild boars. The close affinity between Italian and Corso-Sardinian wild boars observed in the PCA and the maximum likelihood population tree, adds weight to the hypothesis that *S. s. meridionalis* originated from the introduction of Italian wild boars (Albarella et al. 2009; Evin et al. 2015; Groves 1989; Larson et al. 2007). Such a wild origin would explain the close morphological similarities in shape (albeit in miniature) between insular and continental wild boar populations (Albarella et al. 2009; Evin et al. 2015; Groves 1989).

With reference to evidence from zoo-archaeological studies, the genetic data thus hint at the possibility that the first pigs to arrive on Corsica and become feral were of Near Eastern origin, but that both Corsican wild boar and domestic pigs were later replaced or genetically admixed with animals from the Italian mainland. Vigne (1988) had advanced such a hypothesis owing to the sudden appearance of more evolved morphotypes (e.g., with a sub-concave profile that is characteristic of domestic pigs) in the middle of the 3^rd^ millennium BCE. During this era that saw increased trade between Corsica and the mainland, continental domestic pigs may have been brought to the island and crossbred with the local stocks of domestic pigs (Vigne 1988). This is in line with the pattern observed in European domestic pigs as a whole, where the genomic make-up of the first Near Eastern domestic pigs introduced to Europe was almost lost through interbreeding with European wild boars (Frantz et al. 2020).

### Effects of human-mediated interventions

In accordance with our hypothesis, the differing management approaches in pig husbandry in Corsica (seasonal free-range) and in Sardinia (ban on free-range pigs since 2012) were reflected in the extent of domestic pig ancestry in the local wild boar populations. The average proportion of genome-wide domestic pig ancestry was estimated to be significantly higher in Corsican than in Sardinian wild boars. Compliance to the ban on traditional free-range pig farming has been problematic in Sardinia (Mur et al. 2016). Questionnaire-based assessments revealed that nearly all Sardinian and Corsican pig keepers have observed domestic pig x wild boar hybrids, which are generally slaughtered immediately due to their slow growth (Albarella et al. 2007; Jori et al. 2017). Iacolina et al. (2018) previously characterised 12% of Sardinian wild boar samples as hybrids, noting that traditional pig keeping practices likely facilitated hybridisation.

The difference in levels of introgressive hybridisation in Corsican and Sardinian wild boars could also have been affected by differences in pig densities, level of control, or extent of habitat overlap. In Corsica, to meet the growing demands of wild boar game-hunting (for meat or recreation), the intentional hybridisation between domestic sows and wild boars has been reported (Dulat 2020). This concerning practice aims to increase the litter size as a way of increasing the number of wild boars available for hunting and likely represents an important source of introgressive hybridisation in Corsica (Dulat 2020).

### Hybrid identification based on morphological criteria

The classification into pure and hybrid Corsican wild boars based on external phenotypic traits showed a weak correlation with genome-wide domestic pig ancestry levels. While a larger sample size may have increased statistical power, inferred domestic pig ancestry levels differed by less than 5%. Even some of the morphologically pure wild boars were estimated to hold 10% domestic pig ancestry. Local inferred ancestry showed larger differentiation on a few chromosomal regions between pure and hybrid individuals (Fig. 6), which would benefit from further research with larger sample sizes to confirm whether this pattern is representative of other wild boar populations with hybrids. Specifically, the extent to which domestic traits (e.g. SPATA genes) may spread to wild boars should be investigated in more detail. Given our limited sample sizes, we used a binary classification of “pure” and “hybrid” phenotypes and did not differentiate among putative “hybrids” with specific hybrid traits. The classification was based on expert opinion and was thus subject to the interpretation of morphological traits by a single person. An in-depth study should clarify the link between genetic ancestry and specific morphological traits (listed in Supplementary Table S1) to further assess genotype-phenotype relationships. Here, we only focussed on the *MC1R* coat colour gene, given its known effects on phenotypic diversity in pigs (Fang et al. 2009).

The majority of morphologically pure wild boars carried two wild type copies of the coat colour gene, while most domestic pigs were homozygous for European dominant black allele. Putatively hybrid wild boars, which had been categorised based on phenotypic traits (Supplementary Table S1), were found to be more likely to carry the domestic European allele than the pure wild boars. One hybrid boar was heterozygous for a coat colour gene of Asian origin, providing support for the presence of genetic material from modern domestic pig breeds in the local pig breed as well as in wild/hybrid boar.

Given the wide range of phenotypic expressions in Corsican wild boars, morphological criteria alone seem an unreliable tool to detect all hybrid or introgressed wild boars. Limited correlation between morphological traits and genetic markers has previously been described in other species (e.g., Lamb and Avise 1987). In fact, hybrids sometimes display a mosaic of parental phenotypes or can be indistinguishable from parental populations, which is why molecular markers are generally more informative (Allendorf et al. 2001).

### Methodological considerations

Previous studies on genetic introgression from domestic pigs in wild boar populations have mostly focussed on the genetic determination and occurrence of hybrid individuals (Goedbloed et al. 2013; Iacolina et al. 2018) or the presence of domestic gene variants (e.g. for *MC1R*; Dzialuk et al. 2018; Frantz et al. 2013; Nikolov et al. 2017) rather than exploring the amount and genome-wide distribution of introgressed genetic material. Due to the non-random sampling scheme of Corsican wild boars (i.e., we kept an even sample size between morphologically pure and hybrid individuals), summary statistics may not be truly representative of Corsican *S. s. meridionalis* and samples cannot be extrapolated to estimate population-wide hybridisation levels. The putative pure wild boars included in the present study originated from areas where hybridisation, as perceived by farmers and hunters, was supposedly rare.

Genetic divergence, estimated as Weir’s *θ*, was lower between Corsican wild boars and the Nustrale breed than between Corsican and Sardinian *S. s. meridionalis*. This result contradicts patterns of genetic divergence inferred from PCA, STRUCTURE, TREEMIX, and PCADMIX, which all suggested that Corsican wild boars shared closer genetic affinity with their Sardinian conspecifics than with the Nustrale breed. This discrepancy in Weir’s *θ* is likely the result of the observed introgressive hybridisation among Corsican wild boars and domestic pigs. Ignoring introgression may therefore lead to false conclusions, when inferring divergence patterns from *F*-statistics alone.

Any process of SNP discovery carries the risk of ascertainment bias when the method yields loci that are not representative of the spectrum of allele frequencies in the target population (Albrechtsen et al. 2010; Helyar et al. 2011). Specifically, an upward bias in genetic variation and divergence estimates may occur when comparing populations dissimilar to the population of the ascertainment panel (Albrechtsen et al. 2010). While the ascertainment panel of the Porcine SNP60 v2 BeadChip included European wild boar and domestic pig breed samples (Ramos et al. 2009), the SNP discovery was largely optimised based on domestic pig breeds. Our results suggested that wild boars were genetically less diverse than domestic pigs in general, and that the Corsican wild boar was less diverse than the Nustrale breed in specific. Although we cannot exclude the presence of ascertainment bias in this result, lower genetic diversity measures were previously observed in wild boar compared to domestic pigs, both when employing the Porcine SNP60 BeadChip (Goedbloed et al. 2013; Iacolina et al. 2016), as well microsatellite loci (Frantz et al. 2012).

The methods that we used to estimate domestic pig ancestry (i.e., ELAI and PCADMIX) require the specification of pure reference populations. The choice and size of reference populations was previously shown to affect results (Barbato et al. 2017; Smeds et al. 2021). Smeds et al. (2021) reported that levels of mixed ancestry stabilised with reference populations of 20 individuals or more, which is why we employed 25 individuals in each reference population. The inclusion of introgressed individuals in the reference populations could have introduced bias, but pure reference populations may be impossible to obtain given the evolutionary history of *S. scrofa*. We used the inferred ancestry levels in STRUCTURE to avoid the inclusion of individuals with clear signs of admixture in our reference populations.

We deduced the amount of domestic ancestry from the STRUCTURE result at *K* = 2. While we assumed that this uppermost hierarchical level would best reflect the domestic pig and wild boar differentiation, the inclusion of diverged domestic pig breed (i.e. Duroc and Mora Romagnola) may make the comparison at *K* = 2 suboptimal. The difference in domestic reference populations likely explains the marked difference in estimated domestic pig ancestry in Sardinian wild boars among STRUCTURE, ELAI, and PCADMIX.

### Wider implications

While introgressive hybridisation seems to have occurred throughout the evolutionary history of *S. s. meridionalis* and traditional farming practices hold socio-economic and cultural values in Corsica, persistent interactions between wild boars and domestic pigs pose several management challenges. Importantly, wild boar and domestic pig interactions were previously deemed responsible for the maintenance and transmission of several infectious diseases, such as Hepatitis E virus, bovine tuberculosis, trichinellosis, or Aujeszky’s disease virus (Charrier et al. 2018; Jori et al. 2017; Richomme et al. 2010; Richomme et al. 2010). However, the role of sexual interactions (and thereby hybridisation) in the transmission of diseases is not fully understood. Improved management practices that minimise contact between free-ranging pigs and wild boars should therefore be implemented before measures as drastic as in Sardinia need to be taken. Of great concern is also the practice of intentional hybridisation between domestic sows and wild boars to increase the wild boar population for hunting purposes (Dulat 2020; Fulgione et al. 2016). Corsica already has a highly abundant wild boar population, with an estimated annual take of 30 000 animals (ONCFS 2018). The spread of artificially selected genes, such as domestic *MC1R* haplotypes, were linked to increased litter size (Fulgione et al. 2016), which could exacerbate the wild boar population management problem.

The rate of hybridisation events is increasing globally due to habitat change and introductions of non-native species (Crispo et al. 2011; Iacolina et al. 2019; Ottenburghs 2021). A growing number of studies demonstrate that hybridisation is an inherently natural process that has played an important role in the evolution of numerous plant and animal taxa (Anderson and Stebbins 1954; Mallet 2005; Stebbins 1959; vonHoldt et al. 2018). Given the commonality of hybridisation in wildlife, perspectives of conservation geneticists are shifting away from aiming to maintain the “pure” genetic integrity of a species, advocating a more flexible approach to dealing with admixture in species conservation (vonHoldt et al. 2018). Notwithstanding this, introgression from a domestic gene pool with artificially selected traits remains a strongly debated management problem (Randi 2008; Trouwborst 2014). In addition to the risk of undesirable (e.g. that increase invasiveness or reduce local adaption) traits spreading into wild populations, there are concerns on how and to what extent the genetic integrity of wild species should to be conserved (Allendorf et al. 2001; Mallet 2005; Randi 2008). Hybrid swarms of Scottish wildcat (*Felis silvestris*; Howard‐McCombe et al. 2021), emergence of herbicide-resistant teosinte (*Zea mays* ssp. *mexicana*) in Europe (Le Corre et al. 2020), and the risk of extinction by hybridisation in the endangered Java warty pig (*Sus verrucosus*; Drygala et al. 2020) are but a few recent examples of the variety of conservation issues caused by introgression from closely related species.

Corsican wild boars and domestic pigs show clear genetic differentiation despite extensive introgressive hybridisation. This result is in line with the paradigm that divergence can be upheld even in the presence of gene flow (Pinho and Hey 2010). There are indications that the evolutionary histories of both forms were shaped by recurrent introgressive hybridisation, facilitated by human-mediated introductions of continental wild boars and domestic pigs. The remaining genetic traces of modern pig breeds (Large White, Duroc) from the 1980s in contemporary Nustrale pigs highlight the long-lasting effects of introgressive hybridisation. To prevent further spread of artificially selected domestic traits, practices such as the intentional hybridisation of domestic sows and wild boars should be stopped. Finally, given the apparent large extent of introgression of domestic pigs into Corsican wild boars, it is not simple to provide specific guidelines on how to deal with hybrid individuals in the wild, beyond efforts to minimise or at least to avoid increases in the rate of hybridisation.

This study has highlighted that livestock management practices can have far-reaching effects on wild populations. Our results validate the importance of molecular markers to formally estimate the potentially damaging effects of domestic introgression into wild populations with the sole reliance on external morphological criteria proving to be an unreliable predictor of genome-wide domestic ancestry.

## Supplementary information

Supplementary Material

## Data Availability

The 48,222 autosomal SNP genotypes for 85 wild boars and 46 domestic pigs (PLINK and TREEMIX file formats) are available from the Dryad Digital Repository 10.5061/dryad.jq2bvq8bb. Mitochondrial haplotypes are available on Genbank (accession numbers MH746786-MH746796).
