# Supplementary material for: Introgressive hybridisation between domestic pigs (Sus scrofa domesticus) and endemic Corsican wild boars (S. s. meridionalis): effects of human-mediated interventions
Source: Heredity (Edinb). 2022 Mar 10;128(4):279–90. doi: 10.1038/s41437-022-00517-1 (PMC8986821; doi:10.1038/s41437-022-00517-1)
Supplement: Supplementary file 1 — Supplementary Material [file 41437_2022_517_MOESM1_ESM.pdf]

SUPPLEMENTARY INFORMATION

Introgressive hybridisation between domestic pigs (*Sus scrofa domesticus*) and endemic Corsican wild boars (*S. s. meridionalis*): effects of human-mediated interventions

Anna Schleimer*, Lorraine Richart, Frank Drygala, François Casabianca, Oscar Maestrini, Hannah Weigand, Chantal Schwartz, Michel Mittelbronn, Alain C. Frantz

*Contact: schleimer.anna@gmail.com

Supplementary Tables and Figures

Suppl. Table 1. Morphological criteria that local expert (Oscar Maestrini) used to classify wild pigs as 'wild boar' or 'hybrid boar' following the same methodology as outlined by Jori et al. (2016). Phenotypes are arranged in order of decreasing perceived importance as indicators. An individual was characterised as 'hybrid' when at least one hybrid phenotype from the first four categories was observed.

Phenotype	Pure wild boar	Hybrid wild boar
Coat colour	Grey-brown zoned, without spots	Black, white, dotted black, white, or red
Body length & weight	High at the front, low at the back, large shoulders and lean thighs, adults of approx. 40kg	Straight back, large thighs, animal stands high, heavier weight (up to 100 kg)
Ears	Small, upright, and alert	Large, floppy ears that fall over eyes
Head	Long, narrow	Wide, large
Snout	Long, narrow	Short, wide
Rump	Flat, compact	High, plump
Foot	Delicate	Fat
Tail	Straight, bushy	Short, kinked

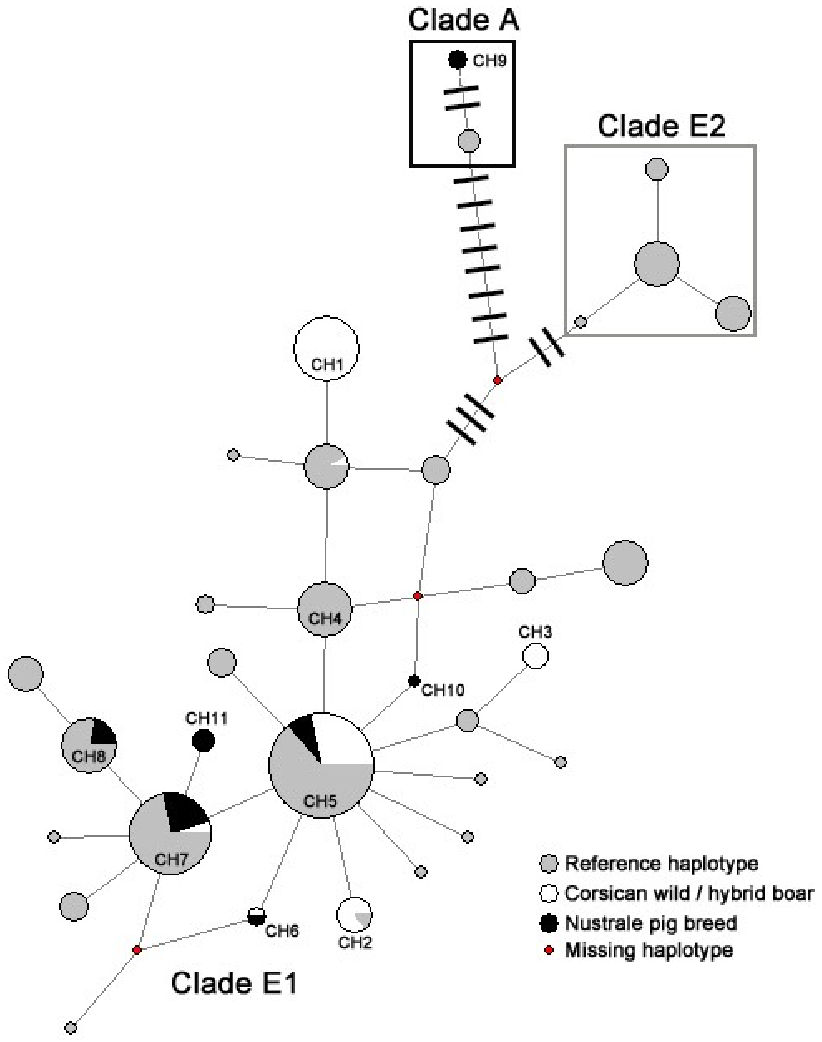

Suppl. Fig. 1. Median-joining network for 32 mitochondrial control region haplotypes from wild boar/domestic pigs. The grey reference haplotypes were identified in a previous study by Scandura et al. (2008). The black and white colours indicate the haplotypes identified in the present study. The overlap of the sequences from the present study and from Scandura et al. (2008) was 83%. Missing haplotypes are indicated by small red dots. The size of the symbol is representative of haplotype frequency.

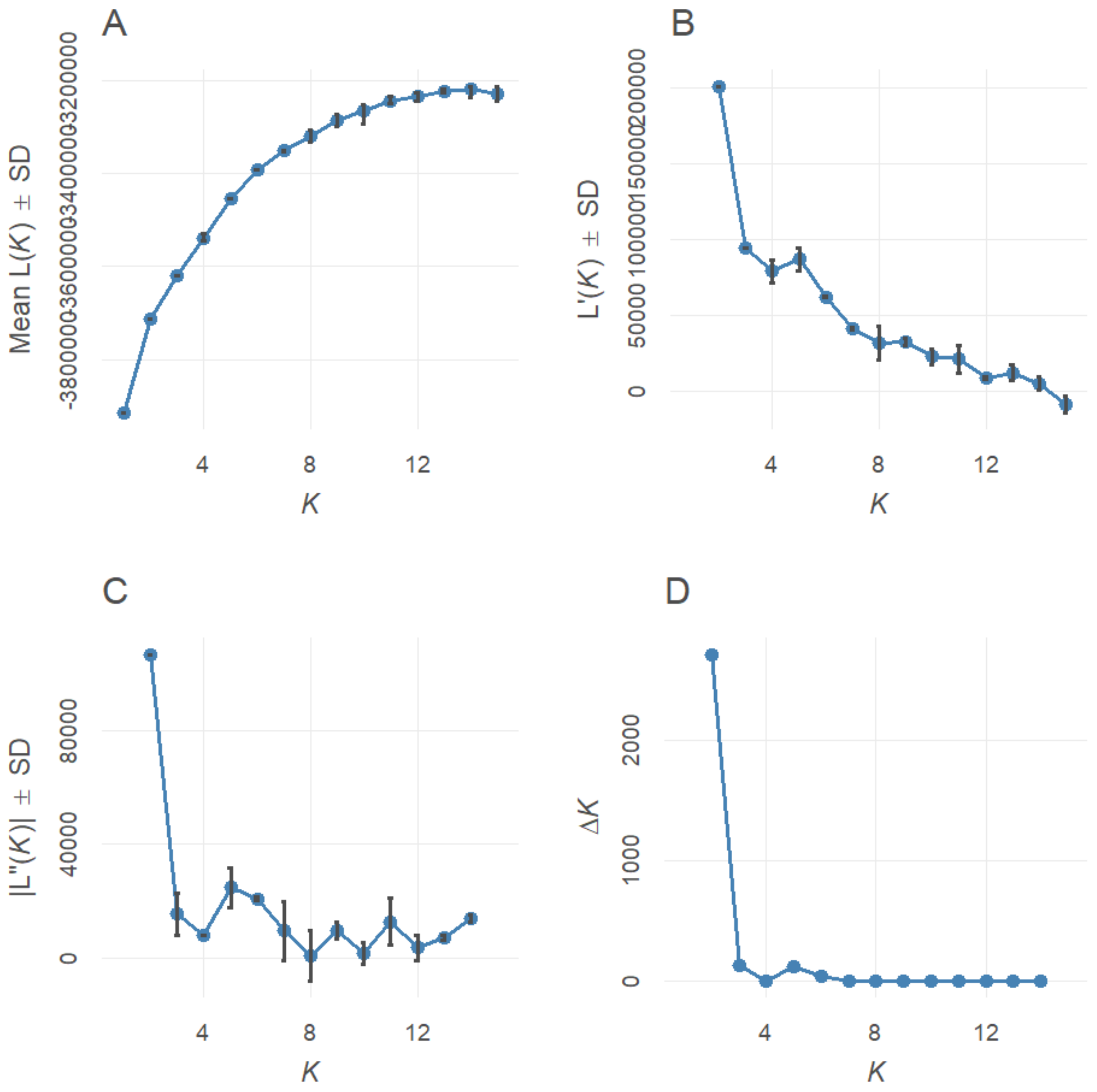

Suppl. Fig. 2. Log $LnP(D|K)$ (L) and derivatives of Bayesian clustering solutions for values of K from one to 15. (A) Mean L(K) (± standard deviation SD) over ten runs for each K value. (B) Rate of change in $LnP(D|K)$ (mean ± SD) calculated as first derivative L′(K) = L(K) – L(K – 1). (C) Absolute values of the second order rate of change of the likelihood distribution (mean ± SD) calculated according to the formula: |L′′(K)| = |L′(K + 1) – L′(K)|. (D) Evanno's ΔK calculated as ΔK = m|L′′(K)|/ s[L(K)]. The uppermost level of structure (here K=2) is inferred from the modal value of this distribution.

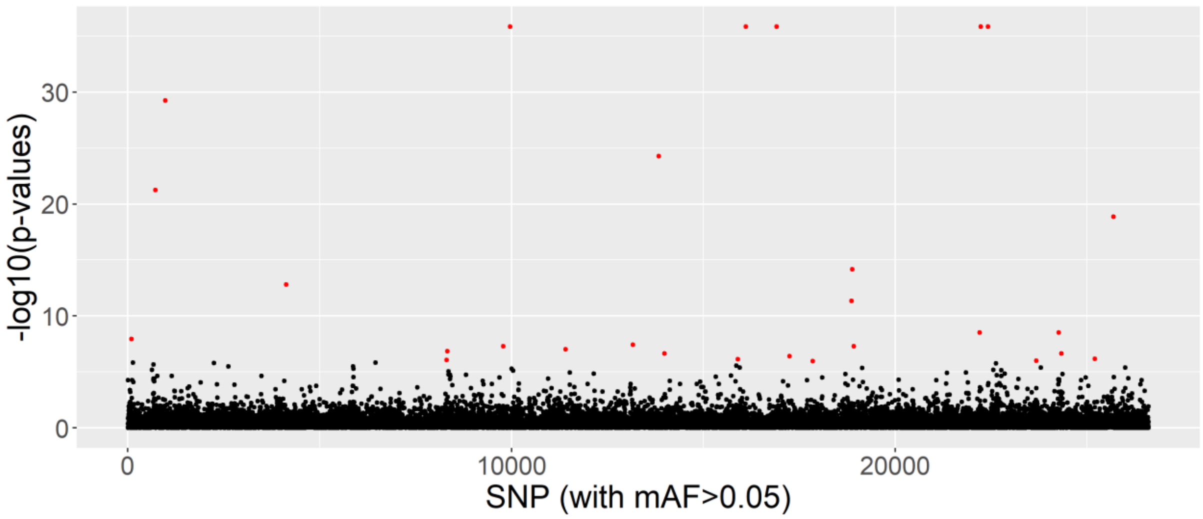

Suppl. Fig. 3. Manhattan plot of the *P*-values of SNPs with the first principal component of the PCA as identified by PCAdapt. The 30 top SNPs having q-values less than 0.1% are displayed in red.

Suppl. Table 2. Outlier loci as identified by PCAdapt among Corsican domestic pig and wild boar samples after application of a false discovery rate at 0.1%. For each loci the SNP name, corresponding accession number (#) and chromosome location (chrom #) are provided. If the locus was associated with a known gene, its name and potential roles, as identified by Ensembl, are given. lncRNA: long non-coding RNA.

SNP NAME	ACCESSION #	CHROM #	CODING?	ASSOCIATED GENE	FULL NAME AND POTENTIAL ROLE
**H3GA0000571**	rs81354717	1	protein-coding	MAP3K4	mitogen-activated protein kinase kinase kinase 4, placenta development, response to UV-C, male germ-line sex determination
**DRGA0000782**	rs80829739	1			
**ASGA0003433**	rs81354796	1			
**MARC0031990**	rs81226673	2			
**H3GA0016069**	rs81383186	5	protein-coding	ENSSSCG00000000399	rhythmic process, lung development, circadian rhythm, morphogenesis of an epithelium, cellular response to DNA damage stimulus
**H3GA0016111**	rs80899191	5	protein-coding	LRIG3	leucine rich repeats and immunoglobulin like domains 3, otolith morphogenesis
**M1GA0008574**	rs81387952	6			
**ALGA0103662**	rs81330691	6	protein-coding	OPRD1	opioid receptor delta 1, adult locomotory behaviour, cellular response to hypoxia, cellular response to toxic substance
**ASGA0032930**	rs80969205	7			
**ASGA0098590**	rs81318877	8	protein-coding	SPATA18	spermatosis associated 18, cellular response to DNA damage stimulus, mitochondrial protein catabolic process
**ALGA0115847**	rs81345194	8	protein-coding	CCSER1	coiled-coil serine rich protein 1
**ALGA0110269**	rs81338505	8	lncRNA	ENSSSCG00000041523	
**ALGA0056816**	rs81428698	10	protein-coding	SPATA17	spermatosis associated 17
**ALGA0057751**	rs81421952	10	protein-coding	CRB1	crumbs cell polarity complex component 1, blood vessel remodelling, visual perception, retina layer formation, eye photoreceptor cell maintenance, cellular response to light stimulus
**ALGA0060892**	rs80866579	11			

**ALGA0062176**	rs80829760	11			
**ASGA0052596**	rs81434447	12	protein-coding	SRP68	signal recognition particle 68
**ASGA0056234**	rs80916343	13			
**ALGA0068352**	rs81441282	13	protein-coding	CMC1	C-X9-C motif containing 1
**ALGA0068598**	rs80892745	13	lncRNA		
**ALGA0083003**	rs80841792	14	protein-coding	C10orf90	chromosome 14 C10orf90 homolog
**M1GA0019738**	rs80794466	14			
**ASGA0068453**	rs80943581	15	protein-coding	ARHGAP15	Rho GTPase activating protein 15, signal transduction, regulation of cell shape
**ASGA0105028**	rs81478796	15	protein-coding	ARHGAP15	
**ASGA0068456**	rs81453232	15	protein-coding	ARHGAP15	
**ASGA0071359**	rs81455887	15			
**ALGA0090171**	rs81458278	16	protein-coding	ARL15	ADP ribosylation factor like GTPase 15
**ALGA0090443**	rs80803543	16	protein-coding	PDE4D	phosphodiesterase 4D, T cell receptor signalling pathway
**MARC0032380**	rs80804246	17	protein-coding	KIF16B	kinesin family member 16B
**ASGA0077907**	rs80836565	17			

References

Evanno, G., Regnaut, S., & Goudet, J. (2005). Detecting the number of clusters of individuals using the software STRUCTURE: A simulation study. *Molecular Ecology*, *14*(8), 2611–2620. https://doi.org/10.1111/j.1365-294X.2005.02553.x

Jori, F., Laval, M., Maestrini, O., Casabianca, F., Charrier, F., & Pavio, N. (2016). Assessment of domestic pigs, wild boars and feral hybrid pigs as reservoirs of hepatitis E virus in Corsica, France. *Viruses*, *8*(8), 1–11. https://doi.org/10.3390/v8080236

Scandura, M., Iacolina, L., Crestanello, B., Pecchioli, E., Di Benedetto, M. F., Russo, V., Davoli, R., Apollonio, M., & Bertorelle, G. (2008). Ancient vs. recent processes as factors shaping the genetic variation of the European wild boar: Are the effects of the last glaciation still detectable? *Molecular Ecology*, *17*(7), 1745–1762. https://doi.org/10.1111/j.1365-294X.2008.03703.x
